# Neural Action Fields for Optic Flow Based Navigation: A Simulation Study of the Fly Lobula Plate Network

**DOI:** 10.1371/journal.pone.0016303

**Published:** 2011-01-31

**Authors:** Alexander Borst, Franz Weber

**Affiliations:** Department of Systems and Computational Neurobiology, Max-Planck-Institute of Neurobiology, Martinsried, Germany; Lund University, Sweden

## Abstract

Optic flow based navigation is a fundamental way of visual course control described in many different species including man. In the fly, an essential part of optic flow analysis is performed in the lobula plate, a retinotopic map of motion in the environment. There, the so-called lobula plate tangential cells possess large receptive fields with different preferred directions in different parts of the visual field. Previous studies demonstrated an extensive connectivity between different tangential cells, providing, in principle, the structural basis for their large and complex receptive fields. We present a network simulation of the tangential cells, comprising most of the neurons studied so far (22 on each hemisphere) with all the known connectivity between them. On their dendrite, model neurons receive input from a retinotopic array of Reichardt-type motion detectors. Model neurons exhibit receptive fields much like their natural counterparts, demonstrating that the connectivity between the lobula plate tangential cells indeed can account for their complex receptive field structure. We describe the tuning of a model neuron to particular types of ego-motion (rotation as well as translation around/along a given body axis) by its ‘action field’. As we show for model neurons of the vertical system (VS-cells), each of them displays a different type of action field, i.e., responds maximally when the fly is rotating around a particular body axis. However, the tuning width of the rotational action fields is relatively broad, comparable to the one with dendritic input only. The additional intra-lobula-plate connectivity mainly reduces their translational action field amplitude, i.e., their sensitivity to translational movements along any body axis of the fly.

## Introduction

When moving in space, an observer creates by its own movement a continuous shift of the images of the environment on the retina. The resulting distribution of motion vectors is called optic flow [1], [2]. Animals make ample use of optic flow information to visually guide and control their course [3], [4], [5], [6]. The neural mechanisms underlying optic flow analysis have been studied particularly well in flies. Their visual system consists of 4 neuropils called the lamina, the medulla, the lobula and the lobula plate. All these neuropils exhibit the same columnar structure as the retina and are retinotopically organized. At the level of the lobula plate, a set of large motion-sensitive neurons is found which are called lobula plate tangential cells. A total of 60 different cells exist in the blow fly *Calliphora vicina* all of which are motion-sensitive [7], [8]. Some of these cells have been also described in *Drosophila* [9], [10], [11], [12], [13]. A large body of experiments suggests that the tangential cells receive their synaptic input from an array of Reichardt-type motion detectors. This algorithmic model for elementary motion detection consists of two subunits which are mirror-symmetrical to each other [14], [15]. Each subunit reads the luminance values measured in two adjacent ommatidia and multiplies them after one of them has been processed (i.e. delayed) by a low-pass filter. The output values of both subunits finally become subtracted. Many characteristics of the Reichardt detector have been verified in the visual responses of lobula plate tangential cells of blow flies [16], [17] and of fruit flies [12], [13]. While it is still unclear which neurons constitute the Reichardt detector, there is good evidence that motion-sensitive neurons with opposite preferred directions provide excitatory and inhibitory input to the dendrites of lobula plate tangential cells in blowflies [18], [19], [20], [21] and fruit flies [12], [13]. In terms of the Reichardt model, these inputs correspond to the mirror-symmetrical detector subunits.

**Figure 1:**
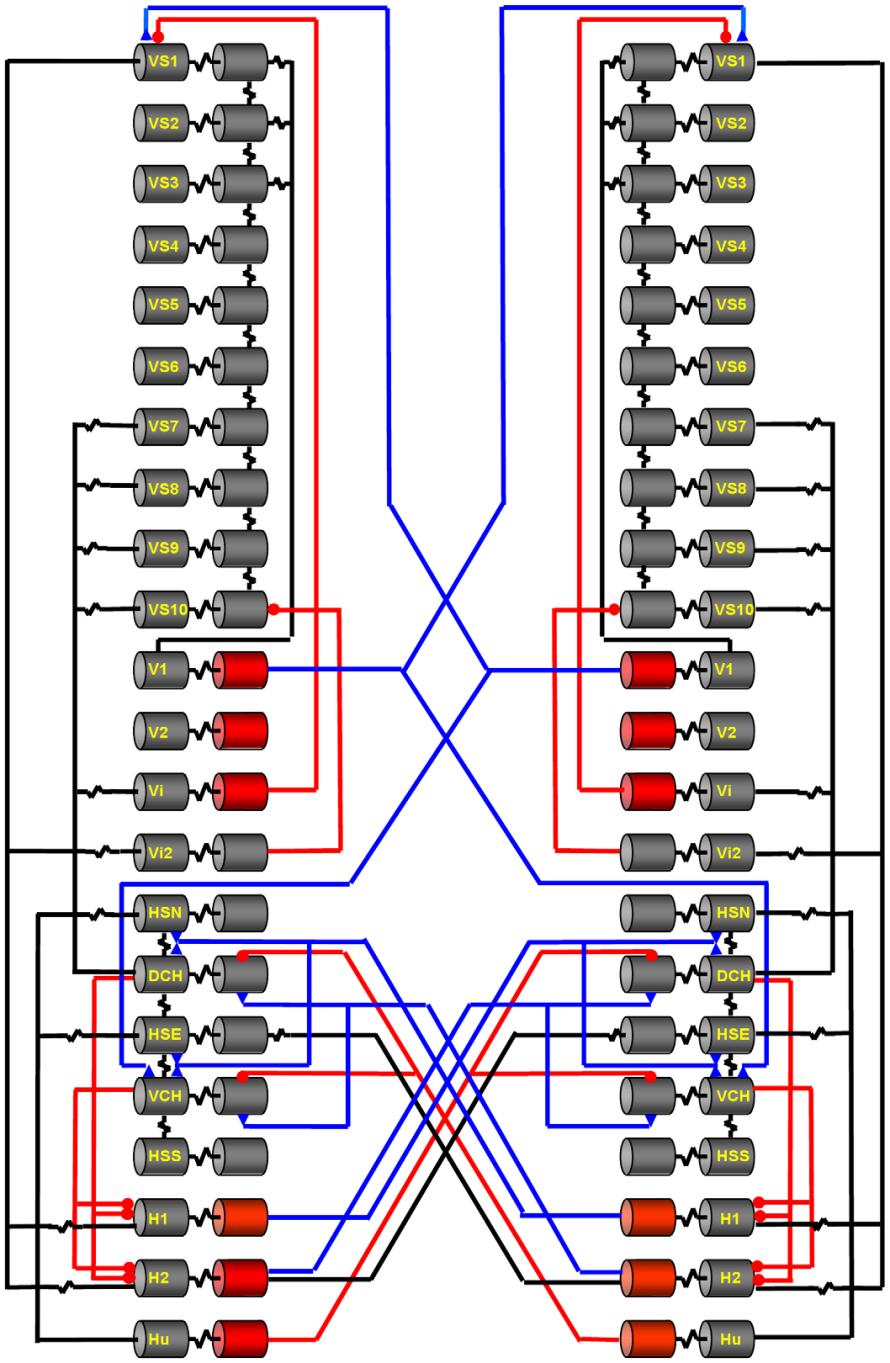
Neural circuit of the lobula plate. For each hemisphere, 22 different lobula plate tangential cells are modeled most of them receiving retinotopic input from local motion detectors (not shown). Each cell is represented by two compartments, one dendritic and one axonal. Compartments are either modeled as passive (grey) or as active, action potential generating elements (red). The connections between the different cells are shown according to their type: electrical synapses, i.e. gap junctions in black, excitatory synapses in blue and inhibitory synapses in red.

After the optic flow is computed by the array of Reichardt detectors, this information now is evaluated by the network of lobula plate tangential cells (Fig. 1). All these cells have large dendrites by which they spatially integrate over various subpopulations of local motion detectors. According to their overall preferred direction, they are grouped into horizontal (H) and vertical (V) cells, respectively (for details see: [7], [21]). Cells of the horizontal system have their dendrites ramify in the anterior layer of the lobula plate. Well studied representatives of this group are the three HS-cells [22], [23], the two CH-cells [24], [25], [26], H1 and H2 [7]. The vertical system comprises V1, V2, Vi, the putative neuron Vi2, and the 10 VS-cells [8], [27]. VS-cells orient their dendrites along the dorso-ventral axis in the posterior layer of the lobula plate. VS-cells are numbered sequentially according to the location of their dendrite from most lateral (VS1) to proximal (VS10). Most tangential cells (HS- and VS-cells) respond to visual motion in a graded way: In response to motion along their preferred direction, they depolarize, and this depolarization is superimposed by action potentials of irregular amplitude [28], [29]. In response to null direction motion, they hyperpolarize. However, in particular those that project their axon into the contralateral hemisphere like H1, H2 and V1 produce regular action potentials. Passive and active membrane properties of HS-, CH- and VS-cells were investigated by current- and voltage-clamp experiments, accompanied by detailed biophysical modeling [30], [31], [32], [33].

According to the retinotopic lay-out of the lobula plate, the location of a cell's dendrite within the lobula plate is a good predictor of its receptive field center. Thus, the three HS-cells which cover the lobula plate in the northern (HSN), equatorial (HSE) and southern (HSS) part have their receptive field centers in the dorsal, middle and ventral part of the fly's visual field. In a similar way, VS1 which has its dendrite in a most lateral position in the lobula plate, is maximally sensitive to downward motion in the frontal part of the visual field: going along with a shift of the dendrite towards more proximal positions within the lobula plate, the maximum sensitivity for downward motion shifts from frontal to more and more lateral azimuth positions in the visual field. However, when investigating the receptive fields of lobula plate tangential cells in detail, Krapp and Hengstenberg [34], [35] discovered that the receptive fields extend over a much larger area along the azimuth than expected from their dendritic field within the lobula plate. Furthermore, they found that the receptive fields are composed of areas with different preferred directions. Overall, the receptive fields have the appearance of curled vector fields such as an optic flow occurring when the animal rotates around a particular body axis. With each cell having a different receptive field, this finding gave rise to the notion that the tangential cells act as matched filters, responding maximally during certain maneuvers of the fly [36], [37]. This was confirmed experimentally [38], [39].

While this observation puts the lobula plate tangential cells on center stage for visual course control, the question remains of how these receptive fields come about. If acting in isolation and strictly in parallel, the receptive fields of all these cells should be much narrower. In addition, their input from local motion detectors is expected to have a more or less uniform preferred direction given that most of the cells ramify within one layer of the lobula plate only. This apparent contradiction was solved by a series of experiments where the signals of two tangential cells were recorded simultaneously. In these experiments current was injected in one of the cells while the response to the current injection was recorded in the respective other cell [40]. These and other experiments revealed an intriguing network within the lobula plate with most of the tangential cells being connected to each other, within each hemisphere as well as between the two hemispheres [7], [40], [41], [42], [43], [44], [45], [46], [47], [48], [49], [50]. The known connectivity of the tangential cells is illustrated in Fig. 1. Many of these connections are based on electrical instead of chemical synapses. This connectivity was hypothesized to account for the large and complex receptive fields: while one part of the receptive field would be brought into the cell via its dendrite, additional information should arrive at the cell indirectly via its neighbors. Therefore, ablating certain cells within the lobula plate should affect the receptive fields of the remaining ones. Performing such experiments via single cell photoablation in blow flies indeed revealed defective receptive fields of the remaining cells [51], [52]. As a step towards a more quantitative description of the response properties of the lobula plate tangential cells, detailed biophysically realistic multi-compartmental models have been created in the past where the neurons received input from arrays of motion detectors as excitatory and inhibitory input, respectively [30], [31], [32]. These models were later also used in network simulations taking into account the connectivity between lobula plate cells of the horizontal (HS- and CH-cells; [53]) and of the vertical system [54]. However, none of these previous studies took into account the full connectivity between all the various tangential cells. For this reason, no previous modeling study could account for the detailed structure of the receptive fields of lobula plate tangential cells as a result of the network connectivity. This is the goal of the present study where we created a network of lobula plate neurons consisting of all 22 cells per hemisphere that have been described in sufficient detail and where data exist for their connection to other cells. Using this model simulation, we also study the consequences of the different receptive field components for the tuning of model neurons to various flight maneuvers, i.e. the neuronal action fields.

## Results

### Probing the Circuit by Current Injection

**Figure 2:**
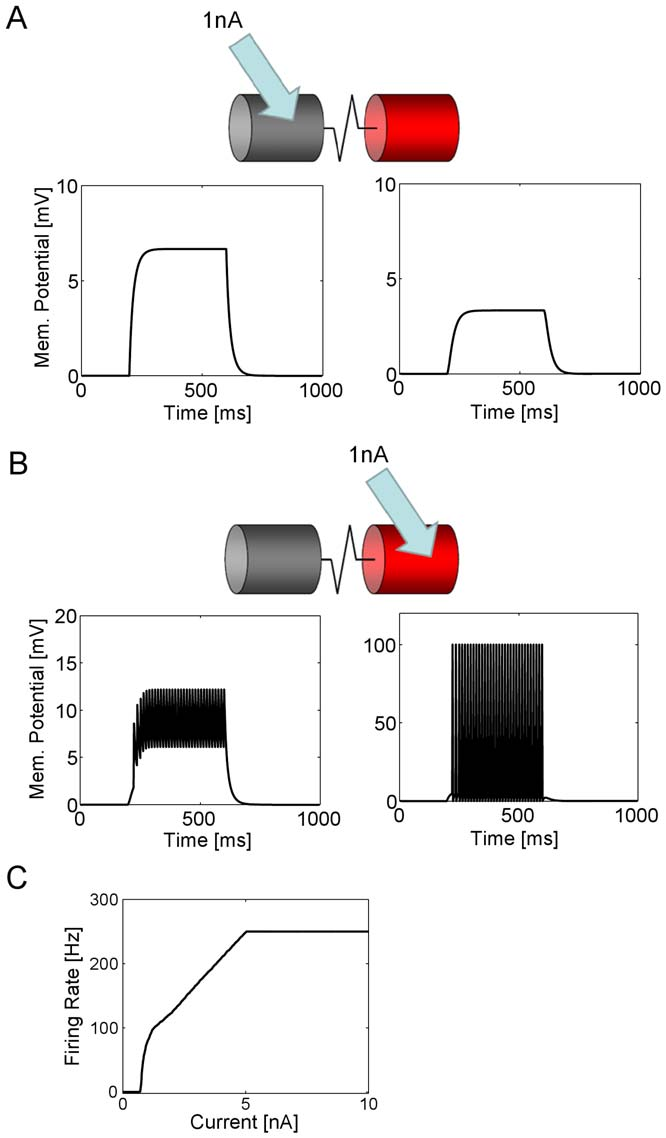
Passive and active properties of isolated model neurons. **a** A current of 1 nA is injected into the dendritic compartment. This results in a depolarization of about 6.7 mV. Roughly 3 mV depolarization is observed in the adjacent axonal compartment. **b** The same amount of current is injected into the axonal compartment directly. This elicits a train of action potentials which passively back-propagate into the dendritic compartment. Due to capacitive properties, the amplitude of the action potentials in the dendritic compartment is much reduced (note different scale on y-axis). **c** Action potential frequency is a function of injected current: After reaching spike threshold, the frequency rises steeply, then has a linear range before saturating at 250 Hz.

To test whether the parameters of the network simulation are appropriately chosen, we performed current injections to probe first the intrinsic properties of single neurons and then their connectivity. In the first series of experiments, all connections within the circuit were shut down, leaving only the electrical connections between dendritic and axonal compartments. Then, cells were injected by a 1 nA depolarizing current. If this current was injected into the dendritic compartment of a spiking cell, the dendritic compartment responded with a graded depolarization of about 6.7 mV, corresponding to an input resistance of 6.7 MΩ (Fig. 2A). This value is by a factor of 2 higher than what is reported experimentally, due to the fact that here cells are considered in synaptic isolation. The axonal compartment responded with a sub-threshold depolarization (Fig. 2A). However, when the same current was injected into the axonal compartment directly, a train of action potentials was elicited (Fig. 2B). Under these conditions, the dendritic compartment is depolarized slightly with a train of action potentials superimposed. The amplitude of action potentials is however much reduced. Using different amounts of current, a current-spike frequency curve was determined. The result revealed the typical Hopf bifurcation at small input currents, followed by a linear range before saturating at 250 Hz (Fig. 2C). This maximum spike frequency follows from a refractory period of 1 time step in the integrate-and-fire mechanism described above given a temporal resolution of 2 msec.

**Figure 3:**
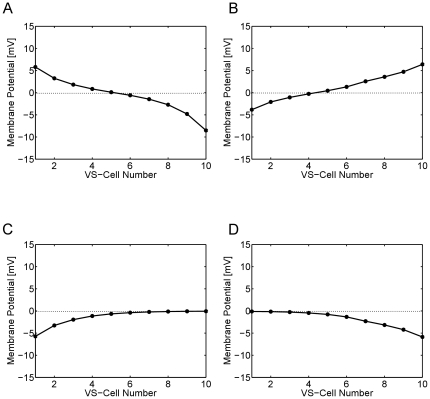
Response of VS-cells (axonal compartments) to injection of 10 nA depolarizing (a,b) and hyperpolarizing (c,d) current into the dendritic compartments of VS1 (a,c) and VS10 (b,c). With increasing distance from the injected cell, the effect gradually decreases. When the injected cell is depolarized, the cell at the opposite end becomes hyperpolarized (a,b). The reversal is not observed when hyperpolarizing current is applied.

A lot of knowledge about the connectivity between different lobula plate cells has been learned from double recordings, i.e. experiments where current was injected into one cell while the membrane potential of another cell was simultaneously recorded. Using the circuit fully connected, we repeated such experiments in the simulation. In the following, however, we will restrict the analysis on the VS-cell network and present the steady-state membrane potential of the cells VS1 to VS10 in response to injection of a constant depolarizing current of +10 nA into VS1 (Fig. 3A) and VS10 (Fig. 3B), respectively. As can be seen, the cells respond the stronger the closer they are to the injected one. The fact can be explained by the sequential electrical coupling between the VS-cells terminals. Moving further away from the injected cell, a reversal of the membrane potential is observed. This is explained by the mutual inhibitory end-to-end coupling via the Vi-cell and the postulated Vi2-cell [46]. When a hyperpolarizing current of −10 nA is injected instead of depolarizing one, a similar but inverted potential distribution is observed as with depolarizing current, except that now, no reversal of the potential is observed at the other end of the VS-cell chain (Fig. 3C,D). All these observations are in close agreement with experimental data [40] as well with previous modeling studies on this circuit [54], [55]. The results confirm that the simulated network, the way it is set up with the magnitude of parameters chosen, is in good agreement with the available experimental data set.

### Receptive Fields

**Figure 4:**
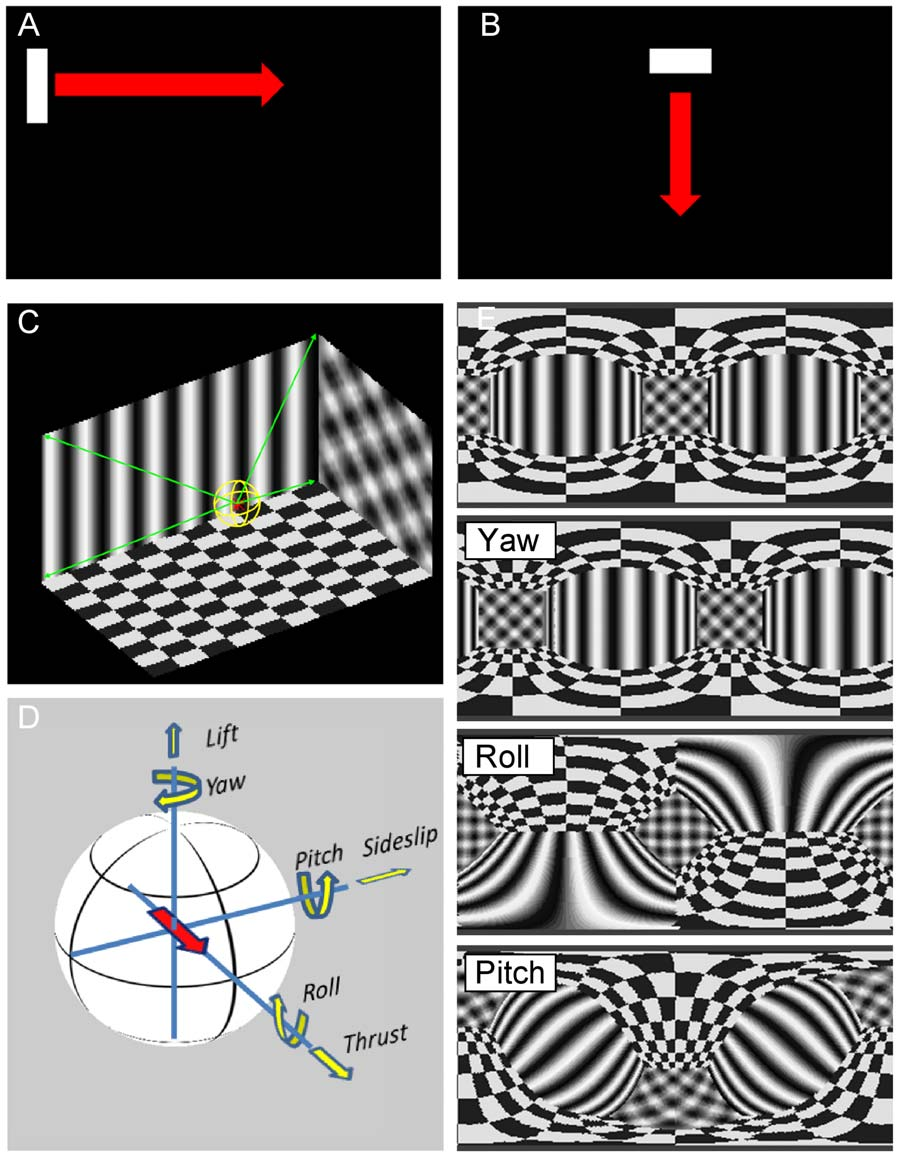
Visual stimuli used to stimulate the lobula plate network. **a,b** To probe the receptive field, a small bar is moved first horizontally to the right at several elevations (a) and subsequently vertically down at several azimuth positions (b). **c** Virtual environment to create full-field ego-motion stimuli. The walls of the virtual room are projected onto a sphere. **d** The sphere can be rotated and translated according to the 6 degrees of freedom. **e** Example snapshots shown in spherical coordinates from image movies obtained for three different kinds of rotations.

**Figure 5:**
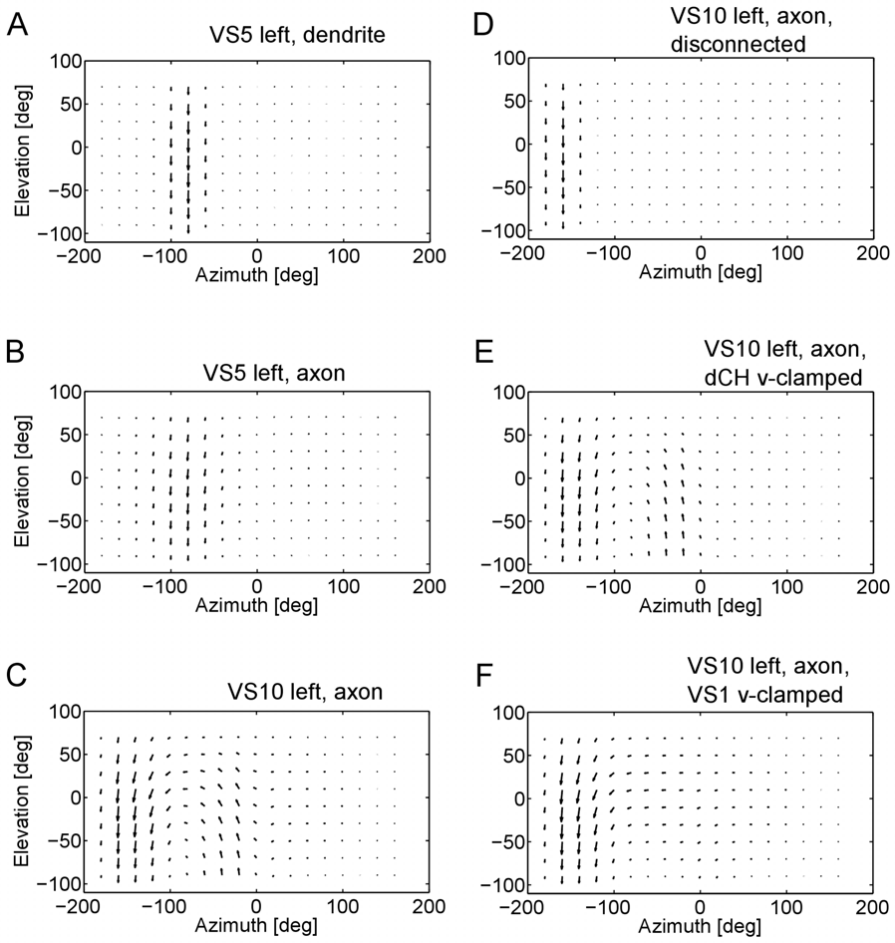
Receptive fields of lobula plate tangential cells. **a,b** Dendritic and axonal receptive field of the left VS5-cell. Due to axo-axonal gap junctions, the axonal receptive field is broadened relative to the dendritic one. **c–f** Axonal receptive field of the left VS10-cell under various conditions. Given full connectivity of the network, the receptive field shows a curl structure with an approximate center of rotation at −90 deg azimuth position (c). With all connections between the cells removed, only the downward sensitivity in the most posterior part of the visual field remains (d). With all connections intact but the left dCH-cell voltage-clamped, the horizontal sensitivity in the dorsal part of the visual field is missing (e). When the left VS1-cell is voltage clamped, the upward sensitivity in the frontal part of the visual field is removed (f).

For probing the receptive fields of the cells, a sweeping bar was used (Fig. 4A,B, see Materials and Methods). It is important to note that the receptive fields shown in the following do not represent the programmed sensitivity fields of the cells plus their preferred direction (see Materials and Methods, Retinotopic Input). Rather, cells in the circuit were subjected to the same procedure that was also applied in experiments on real flies before [13], [39], [56], and, thus, in a sense, were measured. As can be seen in the example in Fig. 5A, the receptive field measured in the dendritic compartment of the left VS5-cell has a rather uniform structure: it is sensitive for downward motion within a stripe of about 30 deg width and, thus, resembles most of what is expected from its sensitivity field as defined in the program code. No additional component is visible that might indicate the network influence. However, this is different from the receptive field as measured in the axonal compartment of the left VS5-cell (Fig. 5B). Although again downward sensitive, the receptive field is significantly broader than the one in the dendritic compartment. Here, a clear influence of neighboring VS-cells becomes obvious leading to a broadening of the receptive field through lateral electrical connections. When moving to the VS10-cell, these effects become most dramatic, as shown in Fig. 5C. The receptive field, as measured in the axon, has a strong curl structure, making it most sensitive for rotational optic flow as occurring during rotational movements of the fly. The receptive field structure has several components: at about −160 deg azimuth position, the receptive field is mainly downward sensitive and, thus, dominated by the immediate dendritic input. At about −10 deg azimuth position, the cell is sensitive for upward motion. This is due to the inhibitory influence it receives from VS1 through the mutual end-to-end inhibition via Vi2. At about −90 deg azimuth position, VS10 is sensitive to horizontal front-to-back motion. This sensitivity is due to its electrical connection with the dCH cell which again receives input from the northern HS-cell, the HSN. At intermediate positions, the local preferred directions assume oblique orientations, in between pure vertical and horizontal orientations. This is despite the fact that there exist only vertically and horizontally oriented local motion detectors. Rather, it is caused by the mixing of input from vertically and horizontally sensitive lobula plate cells.

This mechanistic explanation about how the various parts of the receptive field come about via network interactions within the lobula plate can be vigorously tested in different ways: to reveal the immediate influence of local motion detector input, the cell's receptive field can be probed when all connections between the different lobula plate cells are cut, both electrical as well as chemical synapses. Moreover, specific cells that are likely candidates to be responsible for different characteristics of the receptive field can be voltage-clamped during the receptive field measurement. This has been done for the VS10 cell and the results are shown in Figs. 5D–F. When all cells are disconnected, only the dendritic input survives (Fig. 5D). The cell is now solely sensitive for downward motion in a small stripe of visual space at around −160 deg azimuth position. To test for the influence of the dCH-cell on the horizontal motion sensitivity, the ipsilateral dCH was voltage clamped during the receptive field measurement. The resulting receptive field (Fig. 5E) only shows downward sensitivity at about −160 deg azimuth and upward sensitivity at −10 deg azimuth. To test for the influence of VS1 on the upward sensitivity of VS10 in the frontal part of the visual field, the ipsilateral VS1 was voltage clamped in another run of receptive field measurements. As is shown in Fig. 5F, the upward sensitivity in the frontal part of the visual field is completely absent now.

### Neural Action Fields

Every movement in space can be decomposed and described by 6 parameters (see Fig. 4D). These are the three axes of rotation and the three axes of translation. One way of describing the sensitivity of a neuron is to plot its response in a color coded way on a unit sphere. The strength of the response to rotation around a particular body axis is then indicated by the color of the unit sphere at the corresponding location, i.e. where the axis of rotation would intersect with the sphere's surface. In the same way, the response of a neuron to translation along any axis can be visualized on a separate sphere. Since these are the responses of a neuron as a function of a particular action of the animal, we will call these response fields ‘action fields’. The neural response is fully described by its rotational and its translational action field. Please note that in contrast to the flow field and the receptive field which both are defined and represented as vector fields, the action field is a scalar field, i.e. a scalar function of the two spatial coordinates azimuth and elevation.

**Figure 6:**
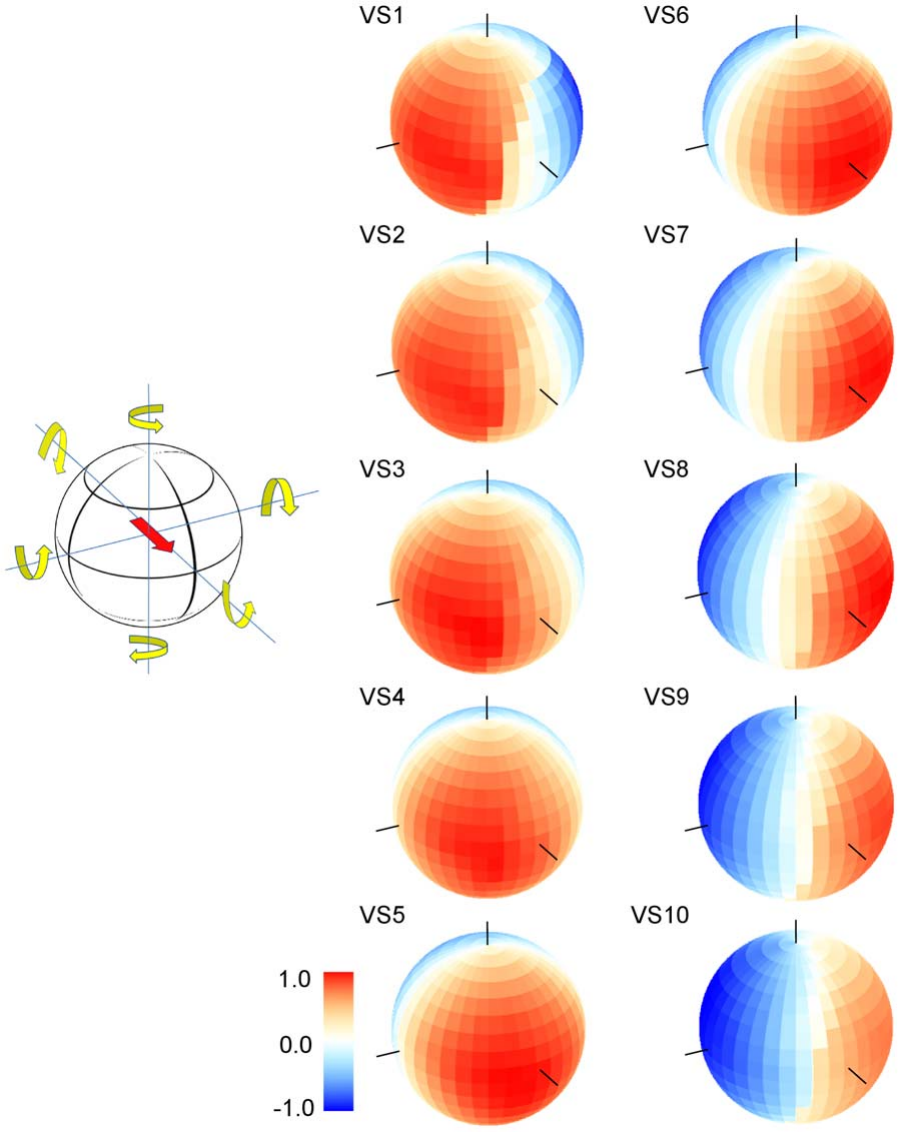
Rotational action fields for all VS-cells, shown in false color code. The orientation of the sphere and the definition of rotation are indicated to the left. The red arrow indicates the viewing direction of the fly. Responses were normalized for each cell to their maximum. The black lines on the spheres indicate the location of the roll, pitch, and yaw axis.

In order to describe the response of any model neuron to all possible movements, we stimulated the lobula plate network with movies resulting from a rotation around and translation along all possible axes in the virtual environment (see Fig. 4E for three rotations) and plotted their responses in the way described. The resulting rotational action fields of all VS-cells located in the hemisphere are shown in Fig. 6. They all turned out to have an individual, single optimum which, however, was rather broad (Fig. 6). As expected from the receptive field (which has a strong downward sensitivity in the frontal part of the visual field), the maximum of the rotational action field of VS1 is roughly at +90 deg azimuth and at 0 deg elevation. Given the definition of rotation (Fig. 6, left), this corresponds to an upward pitch. Rotation around the opposite direction resulted in maximum inhibition. The maximum sensitivity of VS2 is shifted slightly to a more frontal position. Nevertheless, its overall appearance is identical to VS1. This continues all the way through VS3 to VS5 which has its maximum sensitivity in the frontal part of the fly's visual field, i.e. at 0 deg azimuth and 0 deg elevation. Thus, VS5 is particularly responsive to a roll movement of the fly, again as expected from its receptive field with a strong downward sensitivity in the lateral part of the visual field. Continuing through the group of VS-cells, VS10 as its final member is maximally sensitive to rotation around the transverse axis opposite to VS1, i.e. to a downward pitch. Again, this is in accordance to its receptive field with an upward sensitivity in the frontal and a downward sensitivity in the posterior part of the fly's visual field.

**Figure 7:**
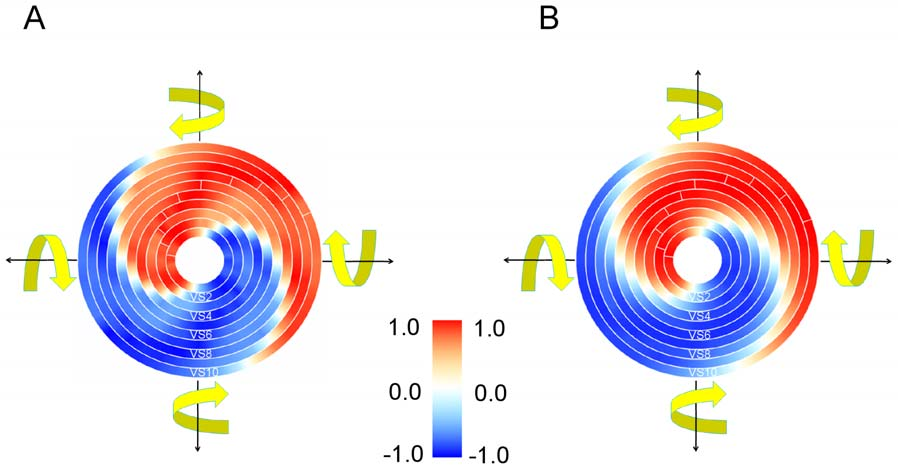
Action fields for rotation within the horizontal plane of all VS-cells, shown in false color code. Responses were normalized for each cell to their maximum. **a** Action fields with all connections of the network intact. **b** Action fields with all connections of the network removed. The action field of VS1 corresponds to the inner most ring, while VS10's tuning is depicted in the outer most ring.

Another way of illustrating the rotational action fields of all VS-cells is to cut through the sphere along the horizontal plane, thus representing the responses of the cells to all rotations within the horizontal plane only. This way, the rotational action fields of all VS-cells can be visualized within a single graph. This is done in Fig. 7A. Here, all features described above become visible again: Each cell has a single optimum, it is rather broadly tuned, and the optimum shifts gradually along the azimuth when going from VS1 all the way to VS10. In the next step, we asked for the importance of the connectivity between the different lobula plate tangential cells for the shape and tuning width of their action fields. We determined the rotational action field for all VS-cells again, this time however with all connections between the different lobula plate cells removed. The result is shown in Fig. 7B. Obviously, the two graphs are almost indistinguishable from each other (compare Fig. 7A with Fig. 7B). It appears that the rotational action field is to a large extend independent from the extra features brought about by the internal connectivity: the direct dendritic input alone is sufficient to produce the same rotational action fields as obtained under conditions of full connectivity with quite elaborate receptive field structures.

**Figure 8:**
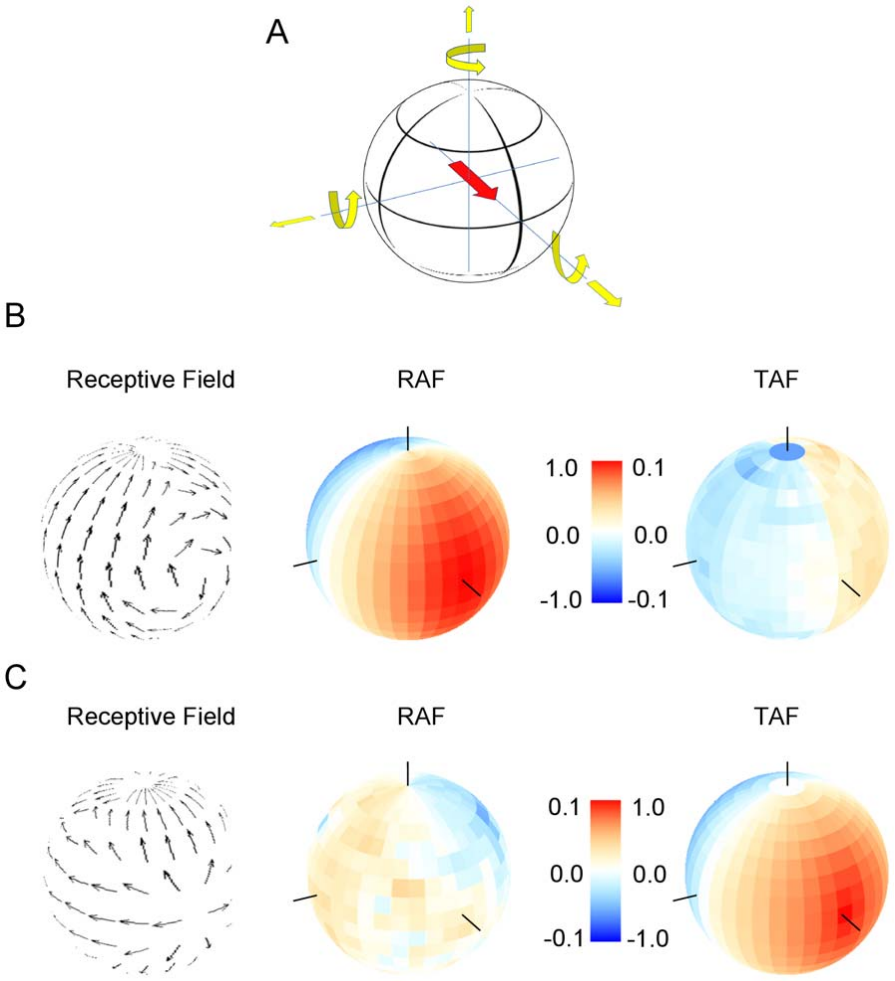
Rotational and translational action field of an ideal rotation (b) and translation (c) sensor. **a** Definition of axes. **b** Receptive field of an ideal roll sensor (left). Its rotational action field is strong and has a maximum in the frontal part of the visual field (middle). Its translational action is extremely weak at all positions (right). The black lines on the sphere indicate the roll, pitch, and yaw rotation axis (middle) or the translation axis for forward, sideward and upward motion (right). **c** Receptive field of an ideal thrust sensor (left). Its rotational action field is extremely weak at all positions (middle). Its translational action is strong and has a maximum in the frontal part of the visual field (right).

In order to understand this result, we constructed an artificial element with the most pronounced tuning that is possible: its receptive field was identical to the flow field resulting from a rotation around the roll axis (Fig. 8B, left). Next, we determined its rotational action field by calculating the average dot product between its receptive field and the flow fields resulting from rotations around all possible body axes (Fig. 8B, center; For details, see Materials and Methods). As expected, the rotational action field has a single optimum located in the frontal part of the visual field. However, it reveals the same broad tuning as was seen in e.g. the VS5-cell. Obviously, the fact that in this case the receptive field covers the whole sphere whereas the receptive field of VS5 reveals only a moderately broad stripe of downward sensitivity in the lateral part of the visual field, has no influence on the tuning width of the two elements. In order to explore that further, we also determined the translational action field of such an artificial neuron (Fig. 8B, right). Here, the response turned out to be virtually zero for translation along all possible axes (note different scale on color bar). This means that elaborate rotational structure of the receptive field has almost no influence on the tuning width of the rotational action field, but greatly reduces its sensitivity for any kind of translation. To complete this exploration, we also designed an artificial thrust sensor by making its receptive field identical to the flow field occurring during thrust movement in the virtual environment (Fig. 8C, left). Calculating its rotational action field in the way described above, the response to rotation around all body axes is negligible (Fig. 8C, center; Again, note different scale on color bar). In contrast, its translational action field (Fig. 8C, right) can be seen to have a single optimum in the frontal position, corresponding to thrust movement which falls off smoothly when deviating from this position along any direction.

While all the above results are obtained from numerical simulations, a deeper understanding of this effect can be obtained from an analytical treatment which is summarized in the following (for a more detailed discussion see Materials and Methods, Analytical Derivation of Action Field Properties). The optic flow or ‘flow field’ is defined as a vector field, representing at each position on the retina the local image velocity. For simplicity, the fly's eye is modeled as a sphere. As can be derived from [2], the rotational and translational optic flow induced by a rotation about the axis **R** and translation along **T** are
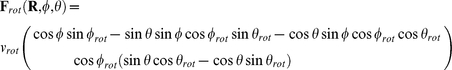
and
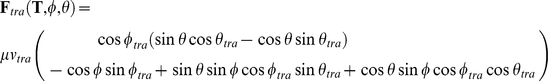
where we expressed the three-dimensional vectors **R** and **T** in terms of spherical coordinates using the parameters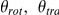 and 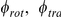 for the azimuth and elevation angles. The rotation and translation velocities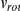 and 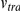 are given by 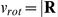 and 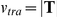. The parameter
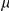
depends on the distance of the sphere to an object, and, for simplicity, is assumed to be constant for each direction.

The receptive field (**A**) is defined again as a vector field, representing for each position in space the cell's local preferred direction of motion (by the vector's direction) as well as its sensitivity for motion (by the vector length). In contrast to the two quantities above, the action field (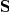) of a neuron is a scalar field. It is defined as the inner product of the flow field and the receptive field.

For a pure rotation around an axis defined by the vector **R**, the action field for the receptive field 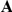 is given by 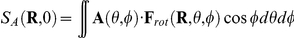
(The term 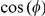 results from the integration over a sphere).

For a pure translation along an axis defined by the vector **T**, the action field for **A** is: 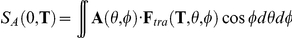



We show below (Analytical Derivation of Action Field Properties) that the translational action field (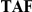) of an ideal linear rotation sensor with receptive field 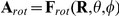 equals zero:
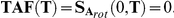



Its rotational action field (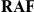) for an arbitrary axis of rotation **V** yields
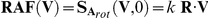
and therefore follows the cosine of the angle between the vector 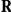 and 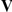. (*k* is a constant independent of 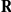 and 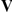). Similarly, the rotational action field with 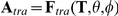 of a perfect translation detector is
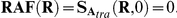



Again, its tuning for an arbitrary translation axis 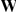 can be expressed as:
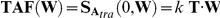



These results also hold if the receptive field does not span the entire sphere (see Materials and Methods).

**Figure 9:**
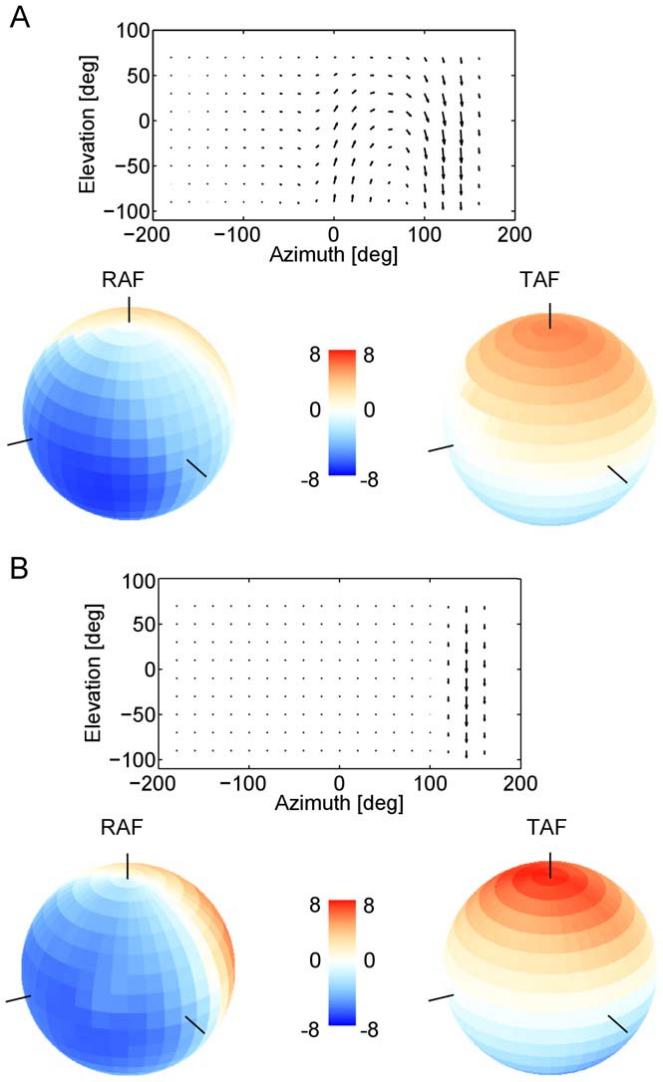
Receptive field and rotational/translational action fields of VS10, given full connectivity within the network (a), and with all network connections removed (b). The internal connectivity has a strong influence on the receptive field and the selectivity of the response for rotation over translation, but no influence on the tuning width for rotations.

These calculations based on the geometrical layout of the different flow fields and the receptive fields of the neuron corroborate the previous observations obtained from model simulations and numerical calculations. We therefore expect the exact structure of the receptive field to have little influence on the tuning width of the rotational action field of VS-cells but to have a strong influence on the strength of its translational action field. In order to test this, we returned to the simulations of the lobula plate network and determined the rotational and translational action field of the cells, with and without internal connectivity. Since the most striking difference under the two conditions was observed for the VS10-cell, the result is shown for this cell only (Fig. 9). With all connections intact, the receptive field shows its typical curl structure (Fig. 9, top). Given that the rotational action field has a single, broad peak in the lateral visual field (Fig. 9A, left), the translational action field is small almost for all axes (Fig. 9A, right). Removing the internal connectivity between the lobula plate neurons leads to a much simplified receptive field (Fig. 9B, top). Nevertheless, the rotational action field is almost unchanged (Fig. 9B, left). However, the translational action field shows a much stronger peak for upward motion (Fig. 9B, right) compared to the fully connected VS10.

**Figure 10:**
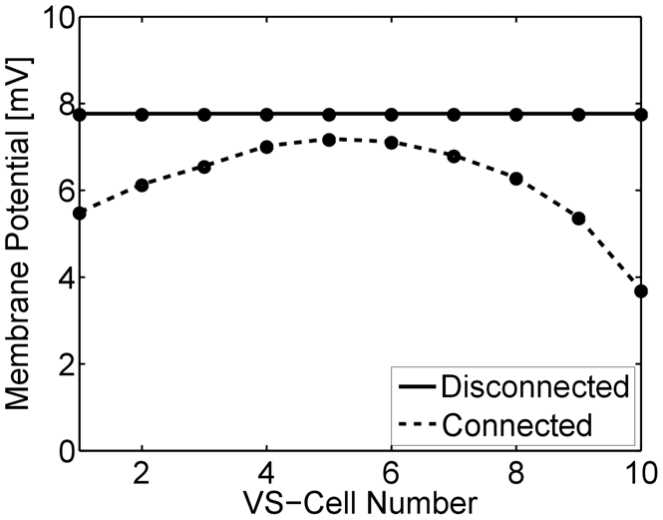
Responses of disconnected and connected VS cells to downward motion. We measured the response strength of all VS cells to a pattern moving homogeneously downward in all parts of the visual field. When disconnected (solid line), all VS cells respond identically. However, when fully connected (dashed line), the response to downward motion is suppressed. The effect is smallest for VS4-6 which are mainly only sensitive to downward motion. In contrast, VS1-2 and VS8-10 exhibit a more rotational receptive field structure and therefore respond weaker to downward motion.

The translation sensitivity of the connected VS10-cell is reduced, since its receptive field strongly resembles a rotational optic flow pattern. However, in contrast to VS10, VS4 to VS6 (see e.g. VS5 in Fig. 5B) are mostly sensitive to downward motion only. According to our theoretical findings, these cells therefore should not exhibit a reduced translation sensitivity. We quantified this by calculating for each VS cell its response to a pattern moving homogeneously downward in all parts of the visual field (Fig. 10). When disconnected, all VS respond the same, since they all receive dendritic input from the same pool of downward-sensitive local motion detectors, only displaced along the azimuth. However, with the connectivity intact, especially VS1 to VS2 and VS8 to VS10, which show a strong rotational receptive field structure, are clearly less sensitive to translation. In contrast, the responses of the connected VS4 to VS6 cells are nearly unchanged compared to their disconnected counterparts.

## Discussion

Based on a large body of experimental data, we created a network simulation of the fly lobula plate where each neuron receives input from a retinotopic array of local motion detectors as well as from all those lobula plate neurons it is known to be connected to. Probing the model with the same type of visual stimuli that were also applied to the real cells, receptive fields were obtained that were close to the ones of their natural counterparts. In particular, cells of the vertical system (VS-cells) displayed, in general, receptive fields that resembled a rotational flow field as elicited during rotation of the fly around a specific body axis. However, in more detail, the receptive fields of model neurons VS4-6 show a more strict downward sensitivity (Fig. 5A) than the ones of their natural counterparts. This might be explained by the particular geometry of the eye and the concomitant orientation of optical axes of the facets [57] which could, in addition to the connectivity between the lobula plate cells, account as well for some features of the receptive field. Furthermore, ablation of VS1 did not lead, as expected from Fig. 5F, to a loss of upward sensitivity of lateral VS-cells (VS8-10) in the frontal visual field [51]. This could be explained by assuming that, other than in our circuit depicted in Fig. 1, all three VS-cells with a frontal dendritic field (VS1-3) provide inhibitory input to the lateral VS-cells independently via the postulated Vi2-cell. As discussed in [51], this would allow VS2 and VS3 to compensate for the loss of the VS1-cell and seems plausible because VS1-3 have highly overlapping receptive fields and collectively are connected to V1 [47], [50].

### VS-Cells have Linear Receptive Field Properties

The simulation of the network formed by the lobula plate tangential cells presented above is biophysically realistic in that it is conductance based: the influence of other neurons and presynaptic local motion elements is not simulated by a mere current injection but takes into account the reversal potential of the respective ion and the concomitant reduction of driving forces. It, thus, is inherently non-linear displaying a characteristic response saturation when large parts of the receptive field become stimulated, either affecting the dendritic input directly or indirectly via network connections. Moreover, the output of active compartments is limited by the refractoriness implemented in action potential generation. Nevertheless, the action fields of VS-model cells are as broadly tuned as predicted from a linear summation expressed as the scalar product between the receptive field and the flow fields occurring during rotation around all possible body axes (see also: [39], [58]). In the same way, the missing responsiveness of the VS10 model cell to translational flow fields can be understood on pure geometric grounds. Thus, by and large, the model cells behave as if they linearly integrate the optic flow weighted by their local preferred directions and sensitivities.

### Optic Flow Processing by the Lobula Plate Network

Our simulation software provides an easy opportunity to disconnect all lobula plate neurons from each other and, this way, allows for investigating the influence of the intra-lobula-plate network connectivity on the cells' action fields. We found that the elaborations of the receptive field caused by the network connectivity changes the rotational action fields in only a negligible way, but strongly reduces the overall amplitude of the neurons' translational action field. Thus, the response selectivity of the neurons for rotational over translational optic flows is increased, but for pure rotations, the selectivity remains the same, compared to the disconnected circuit where all neurons receive input exclusively from their dendrites, and not from other lobula plate tangential cells. One might conclude that, with respect to processing of rotational optic flow, the network connectivity is playing no role at all. This, however, is true only as long as a visual surround is chosen which has a homogeneous contrast distribution. As soon as a more naturalistic environment is used and the responses of VS-cells are considered as a function of time, strong fluctuations of membrane potential are observed in the dendrite, whereas the axon displays a rather smooth and stable response. This effect of connectivity on VS-cell response behavior has been observed in real fly neurons [59], [60] as well in computer simulations which incorporated the VS-cell network only [54], [55], [59]. The results are also in line with studies which considered the joint activity of a population of tangential cells during visual stimulation as occurring during naturalistic movements of the animal during flight [38], [61]. In summary, thus, the network connectivity increases the robustness of representation of rotational optic flow and increases the neurons' selectivity over translational optic flow. Besides the compelling rotational structure of many receptive fields [34], this finding provides further evidence that the lobula plate tangential cells are mainly tuned to rotational optic flow. This is in contrast to area MST in monkey where most neurons where found to be tuned to uniform translations [62], [63].

### Further Processing of Optic Flow in Downstream Neurons

Many of the lobula plate tangential cells synapse onto so-called descending neurons which either transmit the optic flow information to the motor centers of the thoracic ganglion or synapse directly onto a set of neck muscles. Although a systematic investigation on these neurons has only recently begun, the data obtained so far reveal an extraordinary receptive field structure in some of these cells. In particular, neck motor neurons running within the frontal nerve show strongly binocular receptive fields [64] and, thus, seem much more elaborated than the VS-cells in the lobula plate. However, in only a few cases has the connectivity between descending neurons and the respective lobula plate tangential cells been determined [65], [66]. It, thus, seems premature to make some general statements about the representation of optic flow information at the level of descending neurons as compared to the one in lobula plate tangential cells. Here, further work is clearly needed in order to elucidate the action fields of neck motor neurons and how they come about by connectivity. The simulation software used in this study will serve as a convenient platform to incorporate these forthcoming data and, thus, to extend the network of lobula plate tangential cells to the level of descending neurons.

## Materials and Methods

### General

The simulated network comprised a total of 44 neurons, 22 on each side (Fig. 1). These cells have been shown to be extensively connected to each other by various types of electrical and chemical synapses. The experimental basis for the different connections is listed in table 1.

**Table 1:** Experimental basis of connections implemented in the simulation.

Cell-Type	Ipsi or Contra	Connection Type	Reference
VS1-3 -> V1	ipsi	electrical	[47], [50]
H1,H2 -> CH	contra	chemical, excit	[7], [42]
H1 -> HSN, HSE	contra	chemical, excit	[7], [48]
H2 -> HSE	contra	electrical	[41]
Hu -> CH	contra	chemical, inhib	[7], [42]
CH->H1,H2	ipsi	chemical, inhib	[42]
HS->Hu	ipsi	electrical	[42]
HS -> CH	ipsi	electrical	[43]
VS1 -> H1, H2	ipsi	electrical	[44]
V1 -> vCH	contra	chemical, excit	[47]
VS1 -> -> -> VS10	ipsi	electrical	[40]
VS7-10 -> Vi	ipsi	electrical	[46]
Vi -> VS1	ipsi	chemical, inhib	[46]

Each rows describes for the indicated cell pair (Cell-Type) whether the interacting cells are located in the same brain half (Ipsi) or in the left and right lobula plate (Contra), the connection type (electrical or chemical synapse) and the studies describing this interaction (Reference).

All cells within the lobula plate network were modeled by two compartments, a dendritic and an axonal one, which had the following parameter values for leak conductance
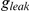
and membrane capacity 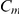, respectively:
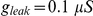


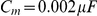



Dendritic and axonal compartments of all cells were connected with a uniform conductance
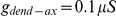



The dendritic compartment of most of the cells received excitatory and inhibitory input from a 2D-array of local motion detectors of the Reichardt type (not shown in Fig. 1). Excitatory and inhibitory synapses had the following reversal potentials relative to resting potential:
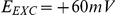


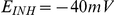



All compartments were modeled as passive RC-elements, shown in Fig. 1 in grey, except the axonal compartments of spiking neurons V1, V2, Vi, H1, H2 and Hu, shown in Fig. 1 in red. In those compartments, an integrate-and-fire mechanism was implemented by setting the membrane potential to 100 mV whenever it grew bigger than a certain threshold, and resetting it to resting potential the next time-step. Spike thresholds were set to the following values relative to resting potential:
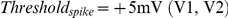


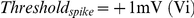


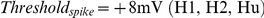



The whole simulation was written in IDL and could be run either from command line or from a GUI. The software allowed for choosing the specific kind of visual motion input (see below), injecting current into any of the cells or clamping a specific cell to resting potential. To easily test the effect of intrinsic connectivity with the lobula plate network on receptive field properties, all cells could be connected or disconnected from each other by a single command.

#### Coupling of cells

Cells were connected to each other either by electrical synapses, shown by a black resistor symbol, or by chemical synapses, symbolized by a blue triangle (excitatory) or a red circle (inhibitory) in Fig. 1. Electrical connections were put into three groups, according to their conductances 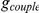: 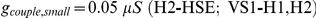


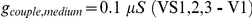


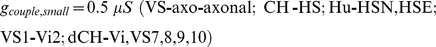



Chemical synapses were modeled as conductances that had a gain of 0.01* µS* except the one of Vi onto VS1 which had a conductance of 0.002* µS*.

### Retinotopic Input

**Figure 11:**
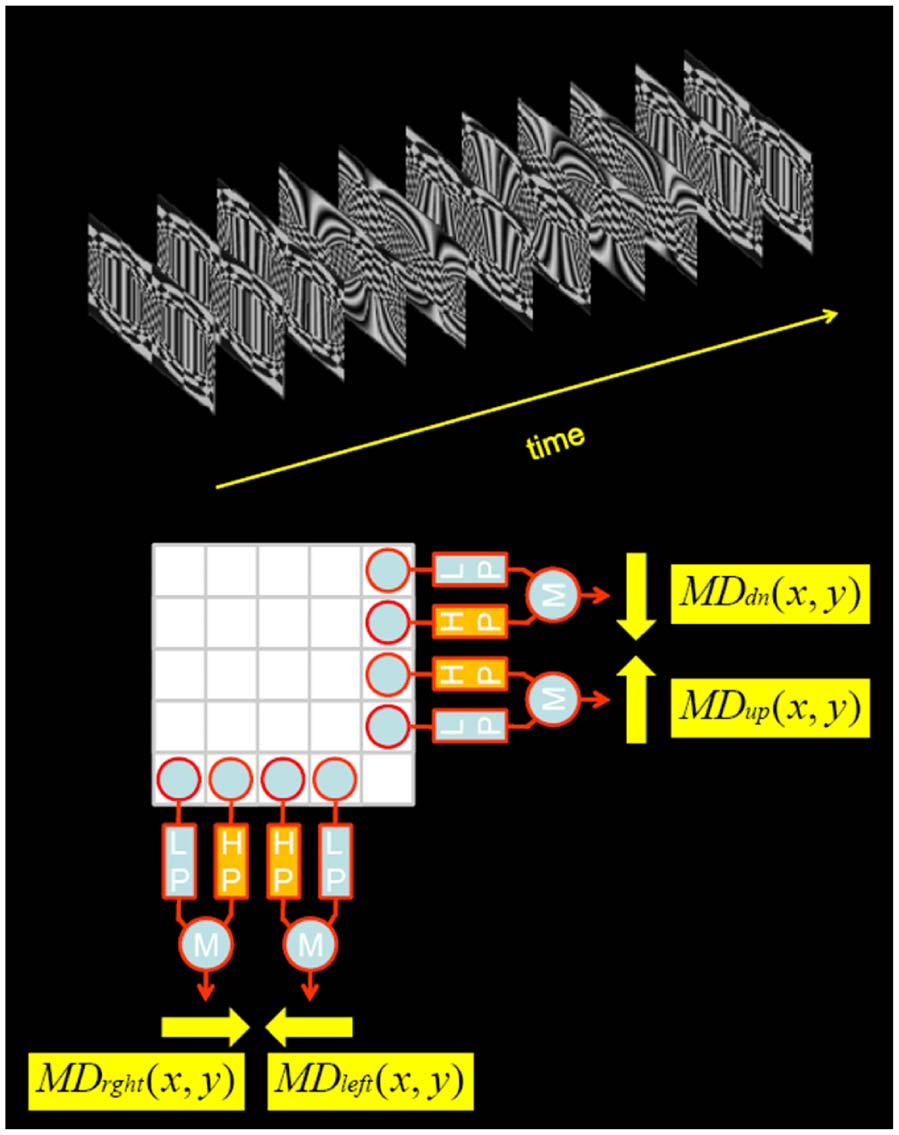
Retinotopic input from local motion detectors. An image sequence is fed onto a retinotopic, two-dimensional array of Reichardt detectors. At each location of the array, 4 different detectors are located: one for rightward, one for leftward, one for downward and one for upward motion. Each detector has one 1st-order low-pass filter and one 1st-order high-pass filter. After passing through the filters, luminance signals of adjacent image pixels become multiplied. The output signals of the multipliers are fed onto the dendritic compartments as conductance values of excitatory and inhibitory synapses, according to the neurons receptive field and preferred orientation.



Any kind of input image sequence was processed by a 2D-array of motion detectors of the Reichardt type (Fig. 11). This array consisted of 4 detector subunits at each location in the image: 2 vertical subunits, one for downward and one for upward motion, and 2 horizontal subunits, one for rightward and one for leftward motion. Two vertically or two horizontally neighboring subunits are separated by a distance (interommatidial angle) of 2 deg [67]. In each Reichardt detector (comprising two subunits), the local luminance value was low-pass filtered and subsequently multiplied with the high-pass filtered luminance value of the neighboring location. The low-pass filter was of 1st order and had a time constant of 20 msec, the high-pass filter was of 1st order as well and had a time-constant of 50 msec [68]. The output values of all detectors were set to 0 when they had negative values. Each cell was given a dendritic sensitivity field 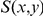 which followed an anisotropic, 2D Gaussian distribution given by the following formula:

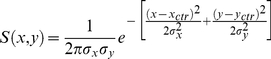
(1)The values for the different cells were set according to those indicated in table 2. Note that in order to account for the inhibitory effect of VS1 onto the lateral VS-cells VS7-10 [46], we postulate an inhibitory interneuron Vi2 the existence of which has not been established yet.

**Table 2:** Receptive fields of the simulated tangential cells.

Cell-Type	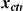	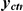	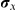	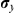	PD	ND
VS1	−10	0	12	60	dn	up
VS2	−26	0	12	60	dn	up
VS3	−42	0	12	60	dn	up
VS4	−58	0	12	60	dn	up
VS5	−74	0	12	60	dn	up
VS6	−90	0	12	60	dn	up
VS7	−106	0	12	60	dn	up
VS8	−122	0	12	60	dn	up
VS9	−138	0	12	60	dn	up
VS10	−154	0	12	60	dn	up
V1	-------	-------	------	-------	------	-----
V2	−80	0	60	60	up	dn
Vi	-------	-------	------	-------	------	-----
Vi2	-------	-------	------	-------	------	-----
HSN	−80	+50	60	40	ftb	btf
dCH	-------	-------	------	-------	------	-----
HSE	-80	0	60	40	ftb	btf
vCH	-------	-------	------	-------	------	-----
HSS	−80	−50	60	40	ftb	btf
H1	−80	0	60	60	btf	ftb
H2	−80	0	60	60	btf	ftb
Hu	−80	0	60	60	ftb	btf

For each cell-type the receptive field center (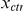,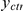), the receptive field width (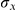,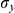) as well as the preferred (PD) and null direction (ND) is specified. See equation 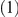 for the formal description of a receptive field. Note that only the values of the lobula plate cells in the left hemisphere are listed. All numbers are given in degree of visual space. Negative x-values (azimuth) refer to the left side, positive ones to the right side of the animal. Negative y-values (elevation) are below the horizon, positive ones above the horizon. Preferred direction and null direction is given in four cardinal directions: dn = downward, up = upward, ftb = front-to-back, btf = back-to-front. If no values are indicated (V1, Vi, Vi2, dCH and vCH), the corresponding cells do not receive direct input from local motion detectors.

The total excitatory and inhibitory input to each cell was then calculated as the sum of the respective motion detector output values weighted by the cell's specific sensitivity. For the VS-cells e.g. which had downward as preferred and upward as null direction, the excitatory and inhibitory visually driven conductances,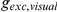 and 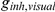, were determined as follows:


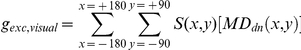


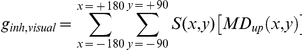



Where the square brackets denote a half-wave rectifier: 
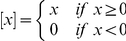



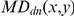
 denotes the output of a downward-tuned motion detector at location 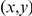. The total excitatory visual input was given a gain of 2* µS*, the total inhibitory visual input had a gain of *3 µS*.

### Visual Stimulation

As visual input to the array of Reichardt detectors, any image sequence could be applied. However, two types of visual input were extensively used in this study. The first one was used to measure the receptive field of the cells in the lobula plate network. It consisted of a small vertical bar of 4 deg width and 8 deg height, that was swept horizontally back and forth at different elevation levels (Fig. 4A). Then, a small horizontal bar of 8 deg width and 4 deg height was swept vertically up and down at different azimuth positions (Fig. 4B). Bar motion was constant at 1000 deg/s. From the response of the cell to this stimulus protocol, a receptive field of this cell was calculated by assigning a vector to each particular position in visual space. The x-component of this vector corresponds to the response of the cell when the horizontally moving bar was passing through this location. The y-component of this vector corresponds to the response of the cell, when the vertically moving bar was passing through this location. This method is identical to the one applied to real fly neurons to measure their receptive fields [39].

A second type of stimulus was used to determine the response of the different cells within the lobula plate network to various large-field motion patterns as occurring during ego-motion of the fly. For that, we created a virtual environment (Fig. 4C). A sphere could be translated and rotated within a box at any speed, around and along any axis (Fig. 4D). The axis as well as the speed could change at any point in time. Typically, however, the speed of rotation or of translation was held constant. The box was decorated with different wall papers. At each time step, the visual pattern was projected onto the sphere and stored as part of an image sequence. The three examples of such motion patterns are rotations around the vertical body axis (‘Yaw’), around the longitudinal axis (‘Roll’) and around the transverse axis (‘Pitch’). Starting from the default orientation with the fly looking at the front wall (top image in Fig. 4E), the different images in Fig. 4E represent a snapshot after 45 deg of rotation has been completed. All images are displayed in polar coordinates. Note that the patterns here are just used for illustrative purposes. For determining preferred rotation and/or translation axes of the cells, i.e. their action fields, wall papers on the ceiling, the side-walls as well as on the floor consisted of regular checkerboard patterns with identical geometry. All movies can be obtained from the authors on request.

### Putting it all together

**Figure 12:**
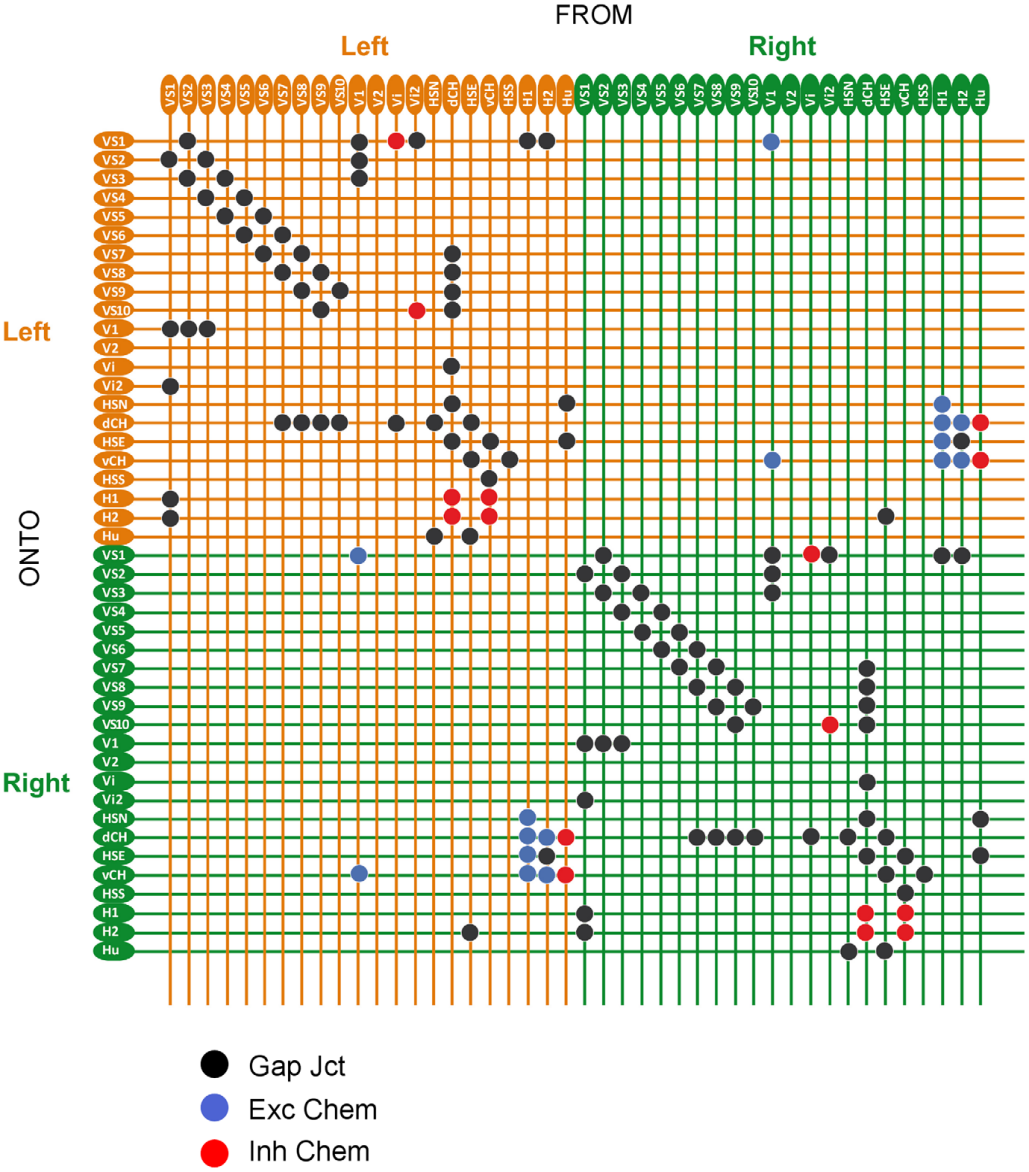
Connectivity matrix of the lobula plate network. The matrix visualizes how the tangential cells within one lobula plate and between the left and right lobula plate are connected. All cells are listed along the rows and columns. A coupling between the cell in row i and the cell in column j is indicated by a dot at the intersection of row i and column j. The coloring of the dots depicts the type of connectivity (black – gap junction, blue – excitatory chemical synapse, red – inhibitory chemical synapse; see legend).

The simulation of the lobula plate network was performed at a temporal resolution Δt of 2 msec. Before each run, a connection matrix 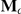 was set up holding the negative values of conductances at the appropriate locations wherever compartments were electrically connected to each other. The diagonal elements of 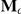 contained the leak conductance of the respective compartment, plus the capacitive conductance, plus the sum of conductances 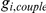 connecting compartment i with all other compartments within the network:
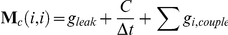



The resulting connectivity matrix is shown in Fig. 12.

Also, an excitatory input matrix 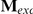 and an inhibitory input Matrix 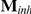 was set up holding the synaptic gains at the appropriate connections between any two cells. When the simulation was started, a specific motion input sequence was selected. At each time t, the output values of the motion detectors were calculated and summed for each cell according to the formulas given above. For each compartment i, this resulted in a total 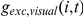 and a total 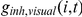. The chemical conductances in each compartment i resulting from activity of other neurons j were calculated as the sum over all N compartments j according to: 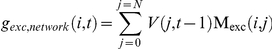


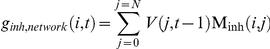
where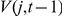 refers to the membrane potential of compartment j at time ‘t-1’.

Conductances resulting from visual input as well as those from other neurons within the network were then added to result in:
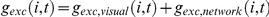


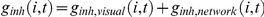



At the beginning of each time-step, the time-dependent matrix 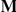 was set equal to 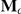. The total excitatory and inhibitory conductances were then added to all the diagonal elements of the time-dependent matrix 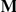:
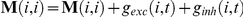



Next, a current vector I was determined holding for each compartment i the following values:
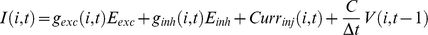



Here,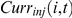 refers to the current injected into compartment i at time t. Note that no leak current appears here, since the leak potential was chosen to be zero. Next, the membrane potential 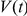 of all N compartments was calculated by solving the matrix equation for 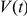:
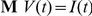



Finally, the membrane voltage 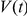 of spiking compartments was set to 100 mV if their spike threshold was crossed, or reset to 0 if the membrane potential was 100 mV previously:
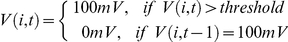



### Analytical Derivation of Action Field Properties

#### Optic Flow, Receptive and Action Fields

A fly whose eye is modeled as a sphere is flying around in an environment. For simplicity, the sphere is centered at the origin 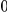
of the coordinate system and its radius has length 1. 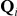 denotes the position of a point somewhere in the surrounding environment. This point is projected onto the point 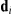 on the surface of the sphere with 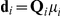, where 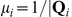
describes the nearness of the fly to the point 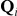. The vector 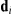 also describes the direction where 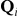 is located. When the fly moves, the point at position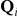 is displaced with respect to the sphere which, in turn, also changes 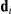, the projection of 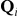 onto the sphere.

According to Chasles' theorem, a movement of the fly can be unambiguously described by a translation along a vector 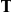 and a rotation about an axis 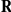 through the center of the sphere. Moreover, a self-motion of the fly can be simulated through a translation and rotation in the respective opposite direction. If the sphere translates forward for an infinitesimal time 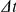, the environment moves backward in the same direction.

The displacement of 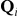 (with respect to the sphere center) due to the translation is given by
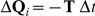



Accordingly, if the fly rotates clockwise around 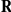, this corresponds to a counter-clockwise rotation of the environment causing the displacement
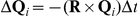



Combining these equations describes the displacement of 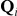 due to an arbitrary motion of the fly. However, a displacement of 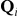 during 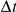 changes the projection of 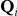 onto the surface of the eye by some 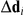. For a motion in the radial direction, i.e. in the direction of 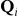, the projection point of 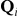 does not change, i.e. 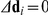. Therefore 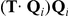 (the projection of 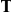
onto 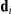) has to be subtracted from the translation yielding the expression:
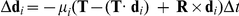
where we used 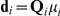. If 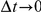, the last equation becomes 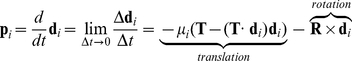
(2)



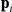
 describes the optic flow induced by a rotation about 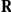 and a translation along 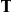. Note that the operation 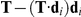 orthogonalized 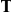 with respect to 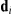. Since the cross-product 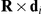 is also orthogonal to 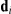, the optic flow 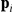 is orthogonal to 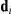.Therefore, although 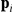 is a three-dimensional vector, it can be unambiguously defined by only two dimensions tangent to 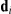. Hence, through multiplication by a 2×3 matrix 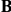, 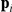 can be projected onto a two-dimensional vector. A convenient choice for B is the matrix
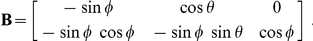



The matrix 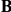 is orthonormal, i.e. 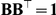. Its rows can be interpreted as a local coordinate system positioned at 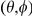 on the sphere with its axes pointing in the direction of the longitude and latitude. Instead by the sub index i, we refer in the following to a location on the unit sphere through the azimuth and elevation angle 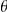 and 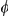. (Here, we follow the convention that the north pole of the sphere corresponds to 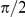.) The values for the azimuth and elevation angle lie in the range 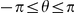 and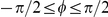, thus specifying the domain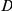. The optic flow at position 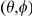 through a rotation and translation about 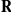 and along 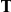 can then be expressed using the two-dimensional vector 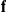 as 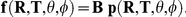



The flow-field 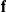 can be decomposed in its translational and rotational component, i.e. 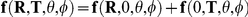 with
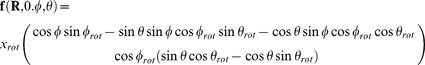
(3)and 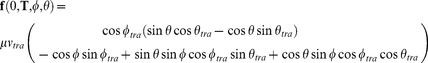
(4)


Here we expressed 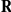 and 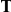 in spherical coordinates, i.e. 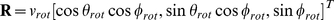
and
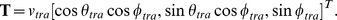



For the following calculations with assume, for simplicity, that the distances to the sphere are homogenous, i.e. 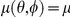 for each location 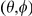.

Optic-flow and receptive fields are vector fields specifying a vector for each point 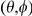 on the unit sphere. In contrast, the action field of a neuron is a scalar field. It is defined as the inner product of the flow field and the receptive field.

#### Optic-Flow Space

Each self-motion can be described through a three-dimensional translation vector 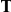 and rotation vector 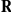. Hence, the space comprising each self-motion is 6-dimensional. Through equation 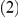 each self-motion is mapped onto a flow-field defined on the unit-sphere. Since equation 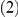 represents a correspondence between the self-motion space and all optic-flow fields, the optic-flow space represents a 6-dimensional subspace in the space of 2-dimensional vectors fields (on the unit-sphere). An orthogonal basis of the optic-flow space is given by three translational optic-flow fields along 3 orthogonal vectors 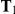, 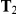 and 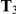 and three rotational optic-flow fields about three orthogonal vectors 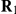, 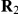 and 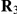. For simplicity, we assume that the vectors 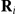 and 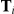 have length 1.

For a proof, we consider the inner product of two arbitrary optic-flow fields with rotation axes 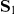 and 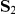 and translation axes 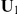 and 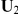 which is given by
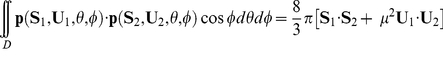
(5)where 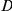 denotes the domain of the unit sphere defined for 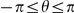 and 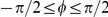. Hence, the inner product of two optic-flow fields equals the sum of the scalar products of the rotation and translation axes. Using spherical coordinates equation 5 can be also expressed as 
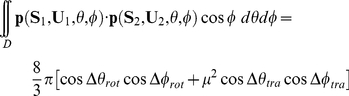
(6)where 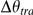 and 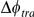 denote the difference between the azimuth and elevation angles of the rotation and translation axis.

From equation 5 all statements showing that the flow fields about and along the vectors 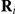 and 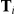 span a 6-dimensional subspace can be directly derived: The inner product between any translational and rotational optic flow equals zero, i.e.
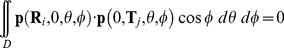
(7)


The inner product between two rotational flow fields about the vectors 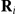 and 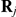 are zero if 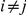
and non-zero for 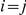. Formally,
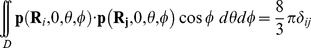
(8)where 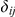 denotes the Kronecker 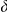-function with 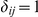, if 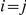 and 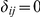, otherwise.

And finally, it can be demonstrated that 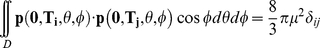
(9)


In the next section, we will demonstrate that this result also holds if the receptive field size of the rotation detector is reduced.

#### Tuning of Small Receptive Fields

We assume again that the receptive field of a considered cell is described by an optic-flow field as induced by a translation or rotation. However, the size of the receptive field is reduced around the center of expansion (or contraction) or the center of rotation. The receptive field size is reduced through introducing two further (non-negative) parameters 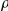 and 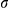 which decrease the integration interval for 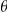 and 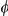 to 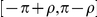 and 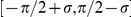 defining thus the reduced domain 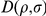 on which the receptive field is defined. 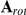 denotes the (small) receptive field of a rotation detector. For simplicity, its rotation center is located at 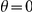 and 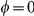, i.e. 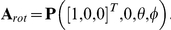 The following results can be generalized to any rotation axis by rotating 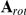. The sensitivity (or action field) of 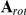 to a translation along 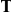 or rotation about 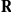 is defined as the inner product of 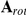 and the optic-flow field. The corresponding action field is denoted by 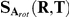.

First, we show that 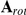 is insensitive to any translation. The sensitivity of 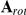 to a translation along 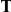
can be written as
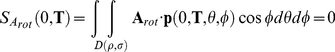
(10)


With 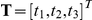 and 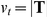. To prove this statement we consider the dot product under the integral which can be written as 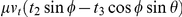. Through integration over 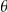 in the interval 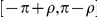 the second term vanishes. Similarly, the first term becomes zero after integration over 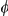 from 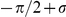 to 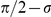.

For the following calculations we introduce the norm of the receptive field 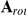 defined as
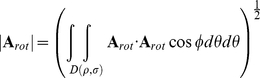
(11)


The dot product under the integral results in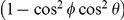. Therefore, the integration of the expression on 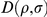 yields
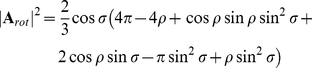
(12)


Using equation 11 the sensitivity of 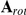 to a rotation about 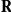 can be expressed as
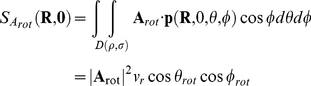
(13)where 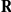 was expressed in spherical coordinates, i.e. 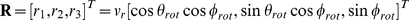. Note that the term 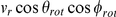 equals the scalar product of 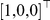 (the rotation axis of 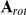) and the rotation axis 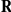.

This result is obtained through integration of the inner product 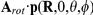 yielding 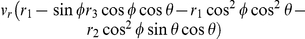 and then expressing 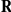 in terms of spherical coordinates.

Analogously to 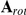, we now consider the tuning properties of a translation-detector 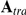 with its center of expansion located at 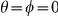, i.e. 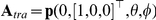. The following analysis can be generalized to any translation vector through rotating 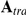. The receptive field size of 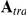 is again specified using 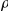
and 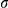. 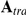 is insensitive to any rotation, i.e.
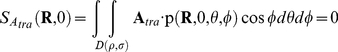
(14)


Setting 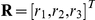, the inner product 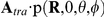 can be written as 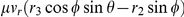. Integrating this expression over 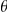 and 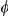 on 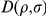 then cancels the first and second term. The norm of the translation detector, 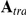 is given by
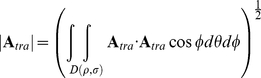
(15)


The dot product under the integral yields 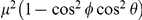. Hence, the norm can be expressed as
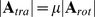
(16)


The translation sensitivity of 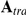 is found to be 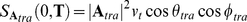
(17)where we set 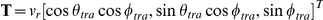. Similarly to equation 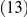 the term 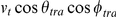 corresponds to the scalar product of the translation axis of 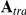 and 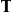.

(All calculations were verified using Maple.)
